# Study of Ni-ZSM-5 Catalysts in the Hydrogenolysis of Benzyl Phenyl Ether: Effects of Ni Loading, Morphology, and Reaction Conditions

**DOI:** 10.1021/acsomega.4c11273

**Published:** 2025-03-17

**Authors:** Raphaël Abolivier, Hans-Georg Eckhardt, James A. Sullivan

**Affiliations:** UCD School of Chemistry, Belfield, Dublin 4, Ireland

## Abstract

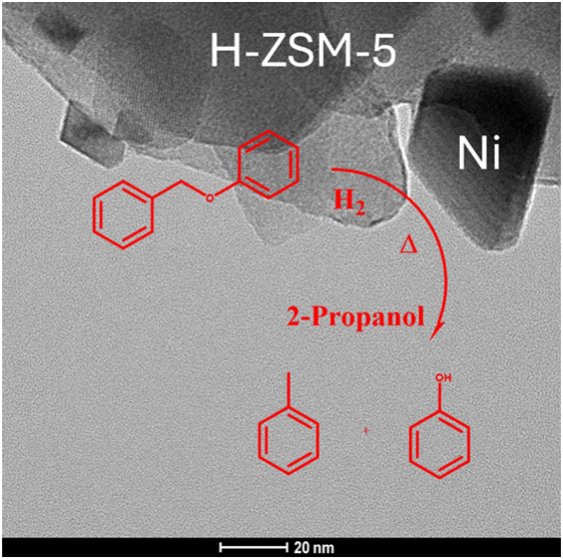

This work investigates the activity of a series of Ni catalysts under a range of reaction conditions for the conversion of a lignin model compound (benzyl phenyl ether) to aromatic compounds. A series of transition metal-based catalysts (Ni-ZSM-5) have been prepared with different metal loadings (5, 10 and 20%) *via* an excess impregnation method. The materials were characterized using power X-ray diffraction (p-XRD), transmission electron microscopy (TEM) and Brunauer–Emmett–Teller (BET) confirming the formation of a set of dispersed, metallic and spherical nanoparticles on all materials. On a 10% Ni-ZSM-5 material, the formation of a set of nanoparticles with a tetrahedral shape was noted. The materials were applied in the cleavage of the ether bond of a lignin model compound (benzyl phenyl ether) *via* hydrogenolysis in a range of organic solvents (1-butanol, ethanol, 2-propanol and pentane) and under two different atmospheres (H_2_ and Ar). 2-Propanol was shown to be the optimal solvent for the reaction confirming its propensity to act as a H-transfer material and the morphology of the supported nanoparticles was shown to have an important effect on the reactivity of the catalysts.

## 1 Introduction

Fossil fuels (*e.g*., crude oil, coal and natural gases) have become an important part of numerous aspects of our lives used in crucial technologies such as energy generation for transportation^1^ and production of electricity^2^ as well as for the preparation of important products and intermediates used in the pharmaceutical^3^ and plastics^4^ industries. This importance relates to their high energy-to-mass ratio, relatively low cost and ease of transportation, storage and transformation.^5^ However, fossil fuels are also an important source of large-scale pollution due the release of previously trapped carbon to the atmosphere at the end-of-life (*i.e*., in the form of Greenhouse Gases).^6^ This increase in CO_2_ levels is in great part responsible for the climate changes that we have witnessed in recent years and should be limited to prevent more dramatic outcomes in the future.

Another limitation to the long-term reliance on the use of such raw material is the depletion and continuously increasing cost of extraction of these.^7^ The extraction of crude oil and natural gas will become more and more expensive as novel and more complex technologies will be required.^8^ This directly affects the viability of the use of this material as a starting material/energy source in numerous industries. While new reservoirs may always be found,^9^ it is clear that we will eventually require alternatives to fossil fuels.

One of the strategies explored to replace fossil fuels and thus to limit the effect of the use of fossil fuels is through the use of biomass-derived feedstocks and biorefining processes.^10^

This technology shares important similarities with current crude oil refining process but relies on the use of renewable biomass as the starting raw material.

Lignocellulosic material is a form of renewable biomass present in the form of microfibrils in the cell walls of all plants (*e.g*., wood, grass, *etc.*) and accounts for 30 to 50% of the mass of plants’ dry matter. It is composed of cellulose, hemicellulose, and lignin, three biopolymers with different structures.^11^ Hemicellulose and cellulose are polysaccharides while lignin is a complex, heterogeneous biopolymer.

The valorization of lignocellulosic material,^12^ can be performed *via* depolymerization of its three main constituents (cellulose, hemicellulose and lignin) followed by the production of compounds that could serve as alternatives to fossil fuel-based analogues in numerous industries. Both polysaccharides (*i.e*., cellulose and hemicellulose) have known valorization pathways within biorefineries, being commonly converted to methane,^13^ ethanol^14^ and/or more complex molecules^15^ such as furfural^16^ and lactic acid,^17^*etc.*

Lignin is composed of three main phenolic subunits bound together by a variety of different possible linkages that are either carbon–carbon bonds or ether bonds (the latter type of linkages being the most common in lignin). The depolymerization of this biopolymer for the production of phenolic molecules involves the breaking of these linkages. The technology for the efficient valorization of lignin *via* the production of platform molecules is not yet mature.^18^

The valorization of lignin *via* the production of value-added compounds is expected to rely on processes that involve catalyzed reactions.^19^ The design of reliable, efficient, cheap and scalable catalysts and processes will therefore be of importance in the implementation of lignin conversion lines within biorefining facilities. The use of transition metals (TM) in catalysis has gained significant interest due to their efficiency in promoting reactions that require hydrogen dissociation/activation such as lignin hydrogenolysis reactions.^20^ Furthermore, their abundance leads to relatively lower cost and ecological impact.^21^ TM-based catalysts still suffer from comparison with their traditional noble metal-based analogues that are more expensive and have a higher environmental impact, but that are usually more efficient. Further work in therefore required for the development of novel and efficient TM-base catalysts for potential future application within biorefinery facilities.

The present work studies the preparation of TM-based, Ni/ZSM-5 catalysts at different metal loadings (5, 10 and 20%) for use in the high temperature cleavage of the ether bond in benzyl phenyl ether, a compound containing an α-O-4 linkage which is a model for those found in lignin *via* hydrogenolysis under a range of different reaction conditions. The results show that reactivity depends on metal particle morphologies, and these in turn depend on the metal loading used. We consider it of interest that a relatively straightforward synthesis method could result in different morphologies depending on the metal loadings chosen. The measured reactivities of the different materials suggest an important effect of the supported nanoparticle morphology and in particular of the presence of Ni(111) exposed facets that efficiently promote the hydrogenolysis reaction. Transmission electron microscopy (TEM) images suggest the formation of Ni nanoparticles with such exposed facets within a specific range of Ni loadings. Probe reactions performed under an Ar atmosphere (rather than the H_2_ atmosphere, commonly used for hydrogenolysis reactions) also showed that a 2-propanol solvent can act as a H-transfer solvent over Ni-containing catalysts. This effect was shown to be sufficient for the promotion of the hydrogenolysis reaction over metal loaded catalysts (with high metal loadings) under the reaction conditions used.

## 2 Experimental Section

### 2.1 Catalyst Preparation

A H-ZSM-5 catalytic support was prepared from the calcination in air of commercially available NH_4_^+^-ZSM-5 at 550 °C for 3 h. H-ZSM-5 supported Ni catalysts were prepared *via* excess impregnation synthesis. The appropriate amount of nickel(II) acetate tetrahydrate (0.85 g for the preparation of 2 g of material at 10% Ni loading) was dissolved in an excess of deionized water. An appropriate mass of the previously prepared H-ZSM-5 (1.8 g for the preparation of 2 g of material at 10% Ni loading) was added to the mixture. The obtained solutions were stirred at R.T. (45 min) followed by an increase in temperature (to 90 °C) for solvent evaporation. The obtained powders were crushed, calcined in air at 500 °C for 3 h and reduced in a flow of 3% H_2_/Ar for 2 h at 500 °C. Three different samples were prepared, with Ni loadings of 5, 10 and 20%.

### 2.2 Catalyst Characterization

The catalysts were characterized by power X-ray Diffraction (p-XRD) on a Rigaku Miniflex 600 equipped with a source of Cu Kα radiation (*l* = 1.54 Å). The scans were conducted in 2θ mode from 10 to 80°.

The samples for transmission electron microscopy (TEM) analysis were prepared by dispersion of a few mg of the samples in 2-propanol. The solutions were sonicated for 15 min and 10 μL of these suspensions were dropped onto Cu-coated TEM grids. The solvent was fully evaporated prior to measurement. The images were recorded on a FEI Tecnai G2 20 Twin Microscope operated at 200 kV.

The N_2_ adsorption/desorption isotherms (BET) were collected on a QuantaChrome NovaWin2 instrument at 77.3 K. The BET specific surface area (SSA in m^2^/g) was obtained from the linear fitting of the data in the N_2_—adsorption/desorption isotherms over the relative pressure range (*P*/*P*_0_) between 0.05 and 0.30 using the Brunauer–Emmett–Teller (BET) equation.

The thermogravimetric analysis (TGA) measurements were performed under an air flow of 60 mL/min (with a balance flow of N_2_ of 60 mL/min) using a Q500 Thermogravimetric Analyzer (TA Instruments). The temperature program consisted of a 5 min isotherm, followed by a 5 °C/min temperature ramp up to 550 °C and another 5 min isotherm.

### 2.3 Catalyst Reactivity Measurements

The reactions were carried out in a high pressure, stirred, 300 mL benchtop 4566 mini reactor (Parr, see Figure S1 in Supporting Information). The temperature (250 °C) and stirring speed (800 rpm) were controlled by a 4848 Parr reactor controller. In a typical reaction, a solution composed of the appropriate amount of benzyl phenyl ether (50 mg) and catalyst (25 mg) in solvent (50 mL) was loaded in the stainless-steel vessel, that was then mounted on the reactor and sealed. The reactor was flushed 3 times with H_2_ to remove oxygen from the vessel and the vessel was pressurized with H_2_ (initial hydrogen pressure, “IHP”: 8.0 bar) and heated to reaction temperature.

After the reaction time (2 h) had elapsed, the heating and stirring were switched off and the vessel rapidly cooled to room temperature using iced water. The reactor was depressurized, and the vessel disconnected from the reactor. An aliquot was collected directly from the vessel using a syringe (equipped with a filter) and transferred to a GC vial for characterization. The GC used was a Varian GC-450 apparatus equipped with a HP-5 column (30 m × 320 μL × 0.25 μL) and an FID detector. The program parameters were defined as follows: an air flow of 400 mL/min, a fuel flow (H_2_ gas) of 30 mL/min and a makeup flow (He gas) of 25 mL/min. The inlet temperature and pressure were 300 °C, 13.2 psi, respectively. The injection volume was 1 μL, injected in split mode (20:1) onto the column.

The catalysts used in these reactions were then collected by vacuum filtration and analyzed postreaction to investigate both changes in structure and the possible formation of carbonaceous deposits on their surfaces during reaction.

For all reactions, the conversion and selectivity to the generated products were calculated using the following equations
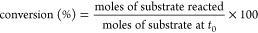

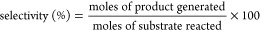


The hydrogenolysis of one molecule of substrate (*i.e*., BPE) generates one molecule of phenol and one molecule of toluene, the selectivities to these two products are therefore expected to be equivalent to one another (and, ideally, equal to 100% for both products). Where a large imbalance was observed between the selectivities to these compounds, this was rationalized in the discussion relevant to the specific data set. Due to the formation of carbonaceous deposits on the catalysts during reaction, and to the occurrence of parallel reactions, including conversion of the expected reaction products to undesired molecules (the levels of formation of these have not been quantitatively measured, however these were arguably formed in very low amounts as suggested by the low GC signals associated with these products) no carbon balance could be achieved. Satisfying selectivities to the molecules of interest have however been obtained for most reactions presented in this work. For the occasions where this was not the case, the byproducts of interest are mentioned, and their potential formation mechanisms are discussed in more detail below.

## 3 Results and Discussion

### 3.1 p-XRD

**Figure 1:**
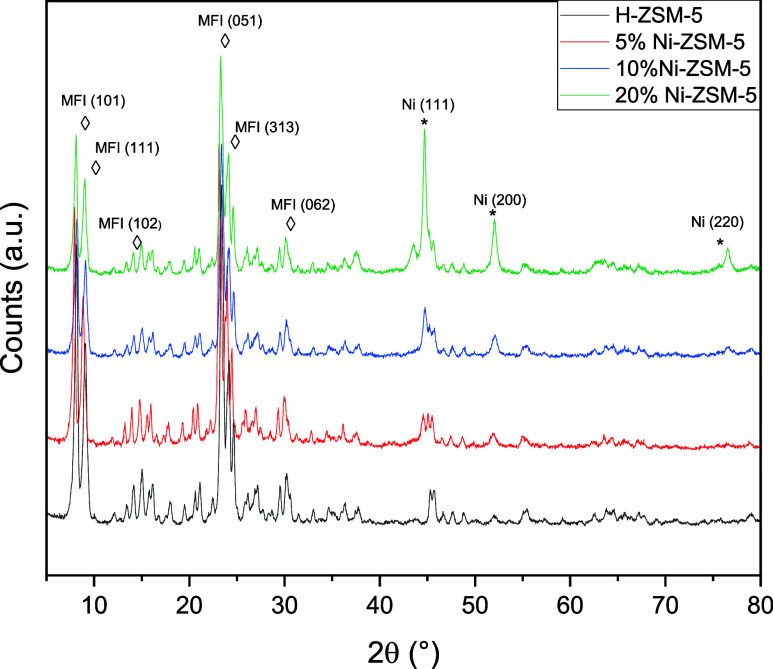
p-XRD profiles of all materials in the Ni/ZSM-5 series and a reference profile of the unmodified H-ZSM-5 material.

The p-XRD patterns of Ni-ZSM-5 series and a reference p-XRD pattern measured for the unmodified H-ZSM-5 material are shown in Figure 1.

A pattern corresponding to that of a tetragonal MFI-type structure (*i.e.*, corresponding to that of a H-ZSM-5 zeolite) with a space group *P*4_2_2_1_2 can be observed in the profiles of all samples. This is confirmed by the presence of (101), (111), (102), (051), (313) and (062) diffraction peaks at 2θ = 8.2, 9.1, 13.9, 23.4, 24.2 and 30.4°, respectively.^25^ Upon the loading of nickel nanoparticles, independently of the metal loading, no shift in either position or intensity of the zeolite support diffraction peaks were observed.

A set of peaks corresponding to a Ni metallic cubic crystalline structure with a space group *Fm*3̅*m* is seen in the profiles of all samples in the Ni/ZSM-5 series. These peaks are attributed to diffraction from the (111), (200) and (220) reflections of the Ni metallic structure with diffraction angles at 2θ: 44.7, 52.1 and 76.6°, respectively26. p-XRD patterns of the 10% Ni/ZSM-5 material have also been collected at the different stages of sample preparation (see Figure S2), showing that the material obtained postcalcination, but prereduction is composed of metal-oxide NiO particles. No evidence for remaining oxide species is seen in any of the profiles of the final materials. As would be expected, the intensity of the nickel structure’s peaks in these profiles increases and the full width at half maximum (FWHM) of these peaks decreases with an increased metal loading. This indicates the presence of more (and larger) Ni particles on materials with higher metal loadings used for their preparation.

### 3.2 TEM

**Figure 2:**
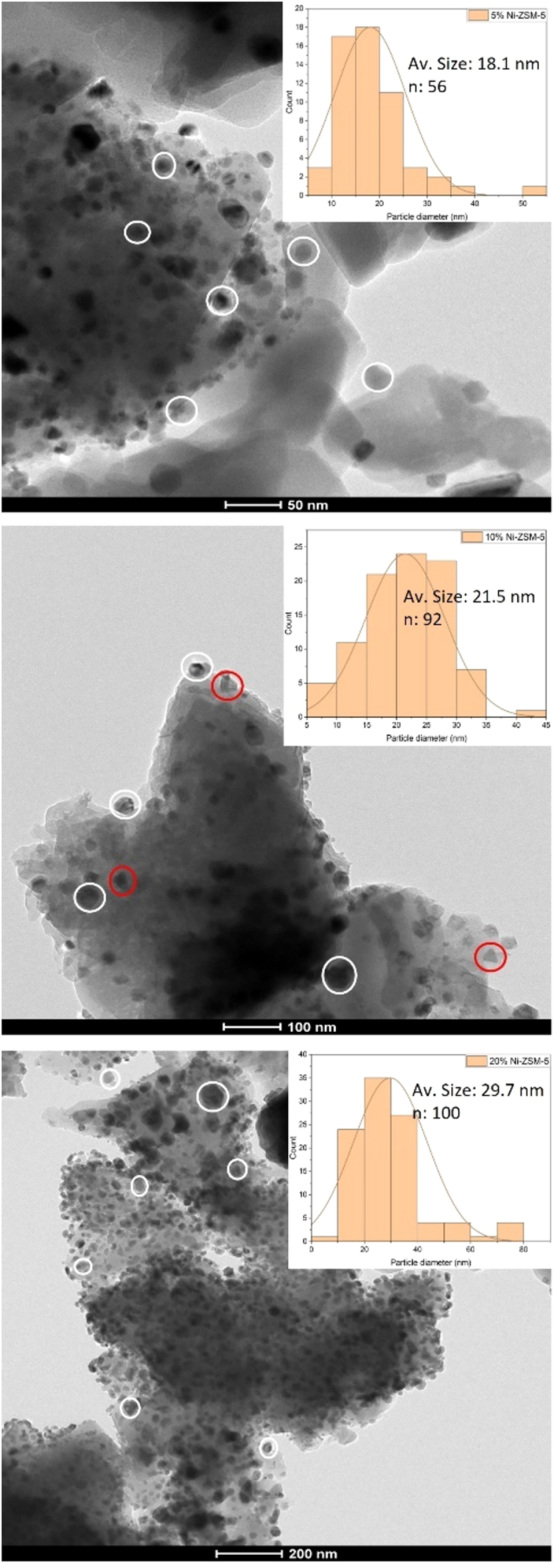
TEM images of materials in the H-ZSM-5 series (5% (top), 10% (middle) and 20% (bottom)). The histograms showing the average particle size analysis is shown as an insert.

**Figure 3:**
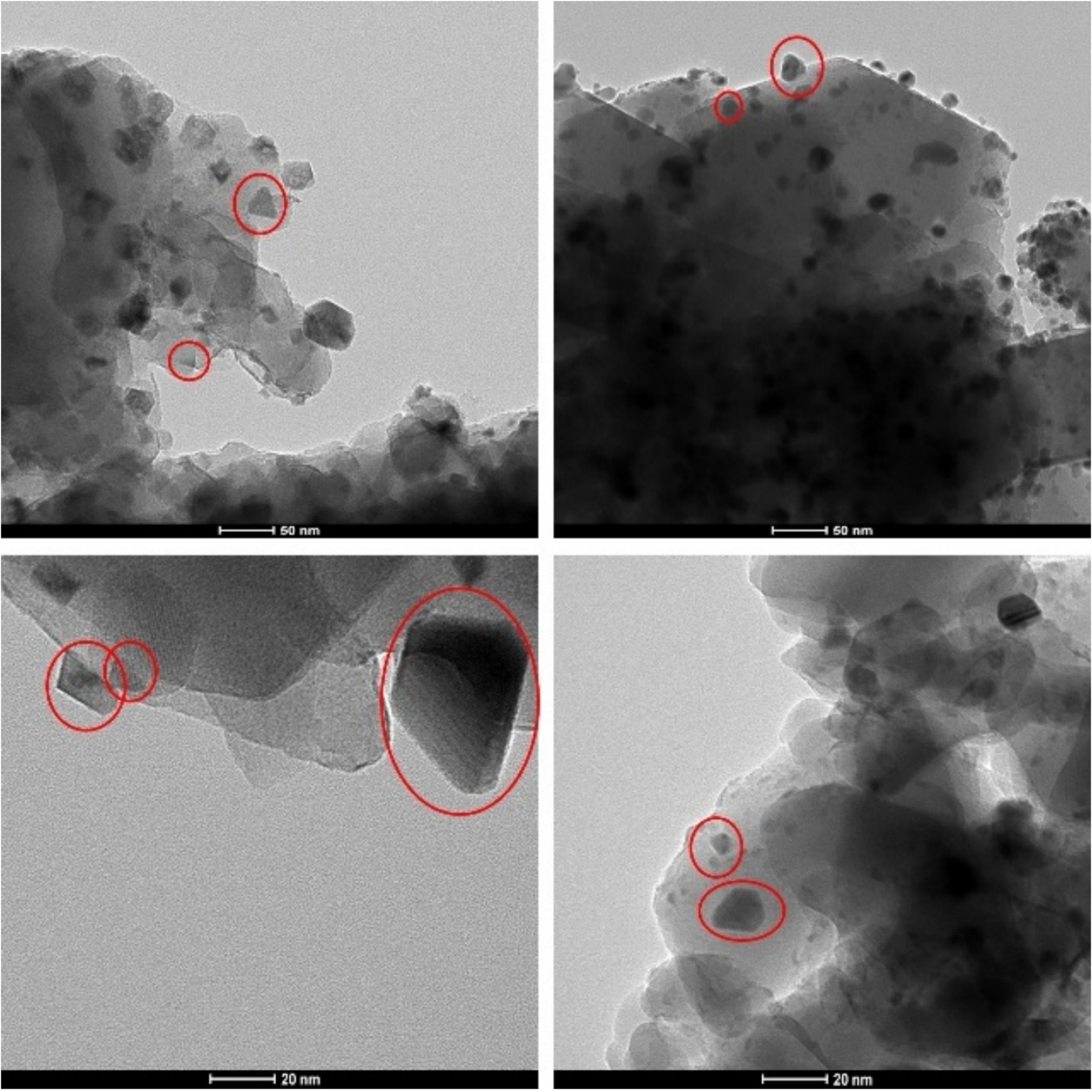
High-magnification TEM images of the 10% Ni/ZSM-5 material. The observed tetrahedral nanoparticles are circled in red.

The TEM images of all materials in the Ni/ZSM-5 series are shown in Figure 2. It can be seen that, for all materials, a set of well-defined spherical nanoparticles (circled in white) can be observed. Interestingly, in the images of the 10% Ni/ZSM-5, nanoparticles with a tetrahedral shape can also be seen (circled in red and shown in more detail in Figure 3). Nanoparticles with this morphology were not seen in the TEM images of either of the other two materials. This shows that the morphology of the H-ZSM-5 supported Ni nanoparticles is dependent on the metal loading used.

A large number of noncorrelated synthesis parameters have been reported to have independent and important influences on the shape and size of the supported metallic nanoparticles. These include, different synthesis methods,^27,28^ different reduction methods,^29^ different solvents,^30,31^ different metallic precursors (*e.g.*, Ni nitrate, Ni acetate or Ni chloride),^32^*etc*. This renders the rationalization of the results observed here, *i.e.*, the formation of Ni nanoparticles with tetrahedral shapes for only specific metal loadings, complex.

However, while a 10% Ni loading led to the formation of a set of nanoparticles with the noted specific morphology (tetrahedral), this was not the case for either the higher (20%) or lower (5%) metal loadings. This could be an indication that under these conditions, uncommon nucleation pathways occurred,^22^ leading to the formation of Ni nanoparticles with unexpected morphologies.^23^

It is also interesting to note that similar observations have previously been reported by Liang et al.^24^ for a set of comparable Ni-ZSM-5 materials also prepared with different metal loadings using an impregnation strategy. They noted that the supported Ni nanoparticles on the catalyst with the median Ni loading had (111) exposed facets and the Ni nanoparticles on the materials with the lowest and highest Ni loadings did not. The presence of Ni(111) exposed facets was shown to affect the materials reactivity with the former material being more reactive than the other two catalysts for the conversion of cellulose to hexitols. No other comparable observations could be found in literature for such catalysts prepared using a simple synthesis method, indicating that this interesting nucleation phenomenon, while not fully understood, has not been commonly considered for structure–property relationships.

**Figure 4:**
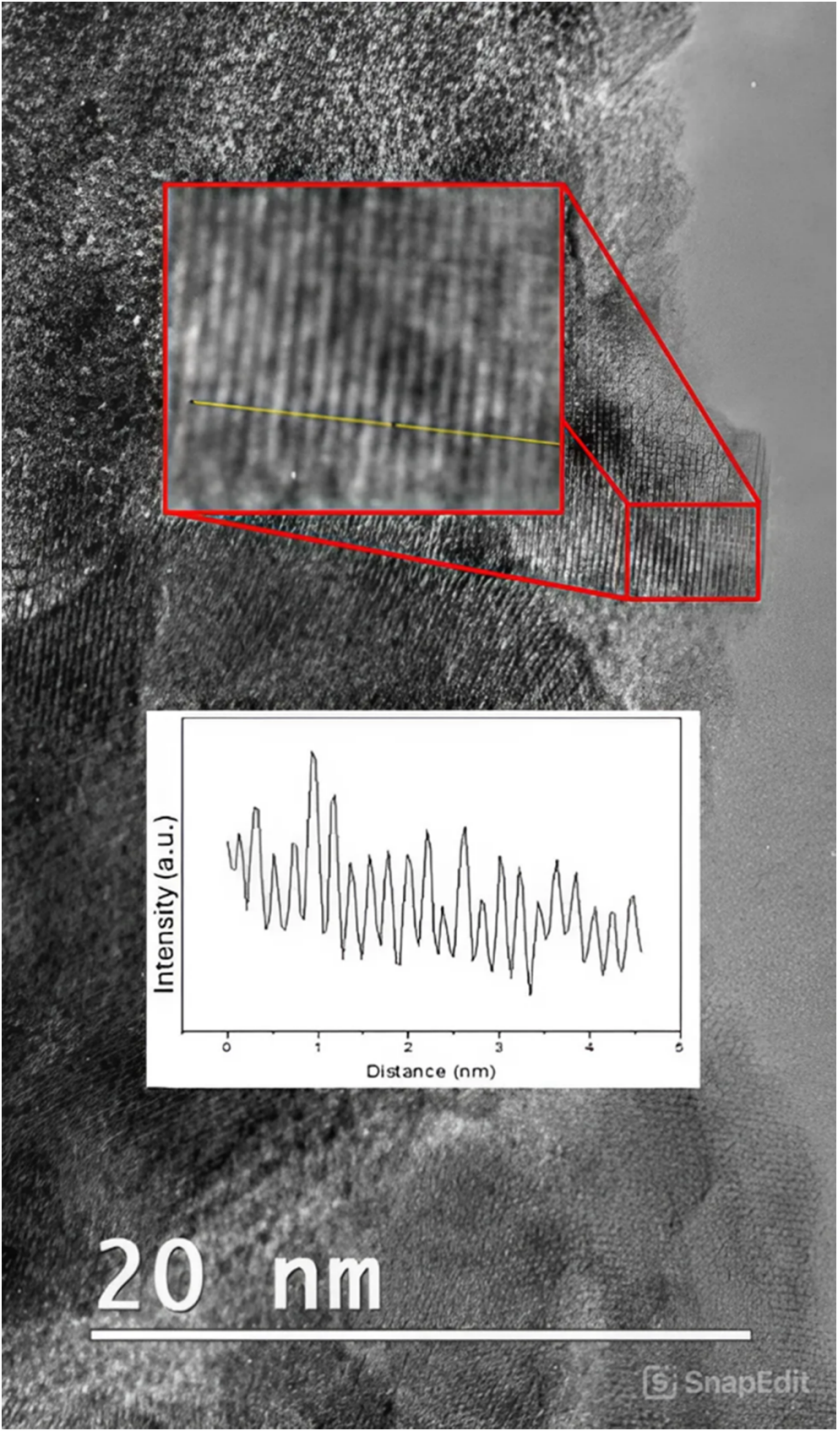
HR-TEM image of the 10% Ni-ZSM-5 material. The profile of the analyzed portion of the image (yellow line) is shown as an insert.

Using Wulff constructions,^25^ it was determined that the tetrahedral nanoparticles observed on the 10% Ni-ZSM-5 catalyst also have exposed Ni (111) facets. This was confirmed by HR-TEM (see Figure 4). The average *d*-spacing calculation was performed on a nanoparticle with a tetrahedral shape. A profile of the intensity of the shade in the recorded HR-TEM image was taken (along the yellow line in Figure 4) and the average distance between consecutives fringes (*i.e*., between the peaks of the plot shown in the inset) was calculated. The average *d*-spacing for this crystalline structure was found to be 0.207 nm, corresponding to that expected from a Ni (111) facet.^26^ This is expected to have implications on the catalytic reactivity of the prepared materials for our study as these (111) facets are known to be highly reactive for reactions that require H_2_ activation such as hydrogenation and hydrogenolysis reactions.^27^

Overall, the nanoparticles are seen to be well distributed over the zeolite’s surface and to have a relatively narrow size distribution for all samples. There is a higher average size measured for materials with higher metal loadings, from 18 nm at 5%, to 21 nm at 10% (including both types of observed nanoparticles) and 29 nm at 20% and this is in-line with the observation made of the p-XRD data. No nanoparticles with sizes lower than 0.56 nm (*i.e*., the pore size of the H-ZSM-5 support)^28^ could be observed in the TEM images of any of the materials, indicating that the nanoparticles were formed, in all cases, on the zeolite support’s external surface and not within the pore channels. This does suggest that they might also block access of reactants to the zeolite’s inner volume.

### 3.3 BET Surface Area

The specific surface areas (SSAs) of all materials in the Ni-ZSM-5 series are displayed in Table 1. As expected, the Ni-loaded materials have lower SSAs than the unmodified support H-ZSM-5 (368 m^2^/g). The Ni-loaded catalysts all have high specific surface areas as expected for metal-zeolite composite materials.

**Table 1:** Specific Surface Areas of all Materials in the Ni/ZSM-5 Series

sample	SSA (m^2^/g)
H-ZSM-5	368
5% Ni-ZSM-5	318
10% Ni-ZSM-5	280
20% Ni-ZSM-5	197

### 3.4 Catalytic Activity

**Figure 5:**
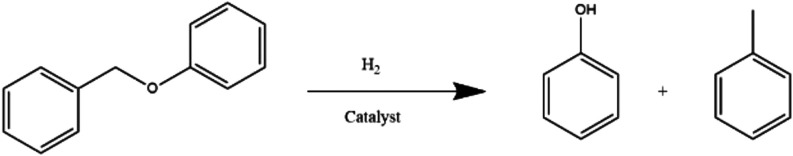
Reaction scheme for the catalyzed hydrogenolysis of benzyl phenyl ether to toluene and phenol under H_2_ atmosphere.

The reactivity of all catalysts was investigated for the hydrogenolysis of benzyl phenyl ether (BPE) to toluene and phenol (see Figure 5).

Preliminary reactions were performed over the catalyst with the highest Ni loading (*i.e*., 20%) to optimize reaction conditions and give insight to the reaction mechanism. First, the hydrogenolysis of benzyl phenyl ether was studied under a H_2_ atmosphere at three different reaction temperatures (200 °C, 250 and 300 °C entries 1,3 and 4, respectively) and another reaction was performed at 250 °C under a lower H_2_ pressure (IHP: 6.0 bar, entry 2). Test reactions were also performed in absence of a catalyst and over the unmodified H-ZSM-5 support to allow insight to the reaction mechanism (entry 5 and 6, respectively). All reactions were performed for 2 h and the results are presented in Table 2.

**Table 2:** Catalytic Results of the Investigation of Different Reaction Conditions for the Hydrogenolysis of BPE in 2-Propanol^a^

*T* (°C)/IHP (bar)	*P*_reac_ (bar)	BPE conv. (%)	toluene sel. (%)	phenol sel. (%)
200/8	19.4	62.1	84.7	68.1
250/6	21.3	84.9	84.1	75.8
250/8	31.9	90.6	82.6	75.8
300/8	51.3	98.8	83.5	74.2
300/8^b^	51.1	14.4	23.2	22.8
300/8^c^	51.4	27.1	6.0	13.7

a Reaction conditions: BPE (50 mg); 20% Ni-ZSM-5 (25 mg); 2-propanol (50 mL); *t*: 2 h.

b This reaction was performed in absence of any catalyst to investigate the thermal degradation of BPE under the selected reaction conditions.

c This reaction has been performed over the unmodified H-ZSM-5 catalyst in order to investigate the role of Ni nanoparticles in the reactivity of the studied material for the hydrogenolysis reaction.

Degradation of the substrate occurred in the absence of a catalyst with a 14.4% substrate conversion under reaction conditions. The two products of interest (*i.e*., toluene and phenol) were generated in comparable amounts to one another but with low overall selectivities (*ca*. 23%) meaning that the desired hydrogenolysis did occur but only at low levels. The only other detected compound for this reaction was p-benzylphenol, (noted in trace amounts).

Regarding a mechanism, the degradation of BPE in absence of a catalyst is expected occur *via* the thermally promoted homolytic cleavage of the substrate generating phenoxy and benzyl radicals^29^ followed by hydrogen extraction from the solvent (*i.e*., 2-propanol, itself converted to acetone in the process) for the formation of phenol and toluene, or to the recombination of the radicals forming low levels of p-benzylphenol alongside large levels of carbonaceous deposit^30,31^ (explaining the low carbon balance obtained for this reaction). This indicates that 2-propanol is not an efficient hydrogen donor solvent under these conditions, this was studied further in the presence of a catalyst, see below.

**Figure 6:**
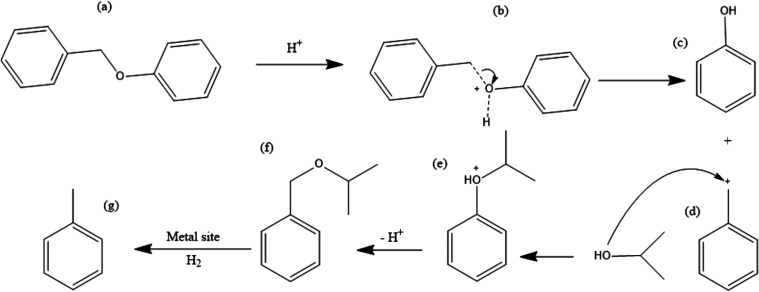
Proposed reaction mechanism for the conversion of BPE to phenol and (isopropoxymethyl)benzene over an acid catalyst (H-ZSM-5).

Interestingly, a higher BPE conversion (27.1%) was obtained in the presence of the acid H-ZSM-5 catalyst, indicating that the zeolite’s Brønsted acid sites can promote substrate conversion. For this reaction (isopropoxymethyl)benzene ((f) in Figure 6), and *o*- and *p*-benzylphenols were generated alongside phenol and toluene. These products were not quantified. The former was generated *via* an activation of the substrate by the acid catalyst through protonation of the substrate’s oxygen weakening the C–O bond,^32^ followed by the cleavage of the model compound, generating phenol and a cation.^33,34^ This cation further reacted with a solvent molecule, regenerating the acid and forming the ether (see Figure 6).^35^ This reactivity could be of interest to produce ester molecules from the valorization of lignin-based molecules over acid catalysts.

As specified in Figure 6, for a reaction performed over a bifunctional metal-acid catalyst and under a H_2_ atmosphere (as is the case for the reactions performed over the 20% Ni-ZSM-5 catalyst), (isopropoxymethyl)benzene can react over a metallic site, generating toluene *via* hydrogenolysis. While toluene is only formed *via* the thermal degradation of the substrate, phenol is a direct product of the acid-catalyzed reaction, explaining the higher selectivity to the former during this reaction.

The formation of benzylphenols also contributed to the overall measured substrate conversions for this reaction, in turn, causing a drop in the apparent selectivity toward both desired products (with respect to the noncatalyzed reaction). The production of the undesired compounds over the H-ZSM-5 materials relates to the acid catalysts’ inability to promote the dissociation of H_2_ (g) molecules or the activation of 2-propanol for the efficient hydrogen transfer reaction that would favor the hydrogenolysis pathway to produce toluene and phenol.^36^

Consistent and high selectivities for the formation of both toluene (*ca*. 84%) and phenol (*ca*. 75%) were obtained at temperatures above 200 °C over the Ni-ZSM-5 catalyst. This indicates that the supported Ni nanoparticles are efficient in the promotion of the hydrogenolysis pathway. No detectable levels of benzylphenols or (isopropoxymethyl)benzene were generated. The lack of production of the former indicates that under these conditions, the radicals formed by homolytic cleavage of the substrate were converted to toluene and phenol by reaction with activated hydrogen species, formed over metallic sites. The latter may have been formed during reaction but would have been further converted to toluene over the metal sites, regenerating 2-propanol as a byproduct. This indicates that the supported Ni nanoparticles play a key role in the hydrogenolysis reaction *via* formation of activated hydrogen species, either through H_2_ (g) activation or through hydrogen transfer reaction involving the solvent. The observed stability in formation of the two products of interest over the range of investigated reaction conditions also indicates that the reaction temperature and pressure do not play a role in controlling the selectivity of the reaction.

A significant evolution of the obtained substrate’s conversion was noted with an increasing reaction temperature from 62.1% at 200 °C (entry 1) to 90.6% at 250 °C (entry 3) and up to 98.8% at 300 °C (entry 4). In the reaction at *T*: 250 °C/IHP: 6.0 bar (entry 2), a lower BPE conversion (84.9%) was obtained than in the reaction performed at *T*: 250 °C/IHP: 8.0 bar indicating that a higher H_2_ pressure also favors the conversion of the lignin model compound, further showing that the activation of H_2_ (g) over the metallic active sites is important for the efficient hydrogenolysis of BPE. In short, higher *T*_reac_ and/or *P*_reac_ were shown to promote the hydrogenolysis of benzyl phenyl ether more efficiently over the same Ni-ZSM-5 material without compromising on the selectivities to the desired products (*i.e*., toluene and phenol).

Satisfying conversion (90.6%) of the model compound was obtained for the reaction performed under relatively mild conditions at 250 °C and IHP: 8.0 bar, these reaction conditions were therefore selected for the rest of the study.

Second, a solvent screening was performed over the same catalyst (*i.e*., 20% Ni/ZSM-5) to investigate the possible role of the hydrogen transfer reaction involving 2-propanol in the hydrogenolysis reaction as suggested by the results presented above. A range of common organic solvents were used, 1-butanol, ethanol, 2-propanol and pentane. The results of this study are shown in Table 3.

**Table 3:** Solvent Screening for the Hydrogenolysis of BPE over 20% Ni/ZSM-5^a^

solvent	BPE conv. (%)	toluene sel. (%)	phenol sel. (%)
1-butanol	28.8	61.9	33.2
ethanol	73.9	85.1	68.8
2-propanol	90.6	82.6	75.8
pentane	98.8	49.2	30.7

a Reaction conditions: BPE (50 mg), cat. (25 mg), solvent (50 mL), *t*: 2 h, *T*: 250 °C, IHP: 8.0 bar.

Of the four selected solvents, the reaction in 1-butanol led to the lowest substrate conversion (28.8%). Interestingly, 1,1-dibutoxybutane and dibutyl ether were the main reaction products in this case. These compounds were formed by a metal-promoted solvent condensation reaction (see Figure S3).^37,38^ Comparable reactivities were not observed in the reactions performed in the other alcohol solvents (*i.e*., 2-propanol or ethanol). This indicates that while 1-butanol can be activated over the Ni active sites, no efficient hydrogen transfer reaction occurs when this relatively large linear alcohol is used as the solvent, but instead undesired condensation reactions are promoted generating H_2_O as the byproduct (*i.e*., leaving no activated hydrogen species for the hydrogenolysis reaction).

The relatively low substrate conversion obtained for the reaction performed in 1-butanol is therefore attributed to this lack of hydrogen transfer reaction. It is also likely that these solvent condensation reactions partially hindered the adsorption of BPE and/or H_2_ by occupying the metal sites, limiting the rate of the desired hydrogenolysis.

Acceptable substrate conversion (73.9%) was noted for the reaction performed in ethanol and no significant levels of solvent condensation reactions were noted. This suggests different behaviors between short (*i.e*., ethanol) and long (*i.e*., butanol) linear alcohols under these reaction conditions with the former allowing hydrogen transfer reaction promoting hydrogenolysis while condensation reactions appear to be preferred for the latter, hindering hydrogenolysis.

Significantly higher conversions were obtained in the other two investigated solvents, 2-propanol (90.6%) and pentane (98.8%). No significant levels of condensation products were detected for the reaction performed these solvents. It was shown that systematic solvent screening using both branched and long/short linear alcohols in hydrogenolysis studies could be of significant interest for the development of efficient development of reactivities relying on H-transfer reactions.

Interestingly the selectivities to both toluene and phenol were significantly lower for the reaction performed in pentane (49.2 and 30.7%, respectively) than for either of the reactions performed in ethanol or 2-propanol.

The difference in selectivity in these reactions was attributed to a difference in solvent-metal interactions. Alcohols such as ethanol and 2-propanol are known to interact strongly with metal nanoparticles,^39^ this is not the case for pentane.^40^

Based on the results obtained from this solvent screening, it is proposed that the lack of interaction between pentane and the metal-based catalyst allowed for, in part, more available actives sites for BPE and H_2_ adsorption (*i.e*., leading to a higher substrate conversion in pentane) but mostly favoring parallel reactions and the formation of undesired products (notably forming cyclohexane with a 28.5% selectivity and benzylphenols in lower amounts, see Figure S4) while the partial occupation of Ni active sites by both alcohol solvents favored the hydrogenolysis reaction, and in turn increased selectivity to the formation of toluene and phenol.

No cyclic product nor any benzylphenol compounds could be detected at high levels for any of the reaction performed in alcohol. This is further indication that the use of solvents that strongly interacts with metals favors preferential hydrogenolysis over hydrogenation of the substrate’s benzene ring possibly due to steric hindrance linked to the coordination of solvent molecules on the metal active sites.^41^

The presence of adsorbed solvent molecules on the metal nanoparticle’s surface is known to hinder π planar adsorption of the model compounds (*i.e*., with a benzene ring parallel to the nanoparticle’s surface) and favor adsorption on the metal nanoparticle *via* the substrate’s oxygen atom.^42^ The former adsorption pathway is known to favor the hydrogenation of the substrate’s benzene ring while the latter is known to favor the direct substrate’s hydrogenolysis.^43^ Overall, these results indicate that BPE can be efficiently converted under these reaction conditions in a solvent that does not interact with the catalyst, however to undesired (in our work) compounds. The selection of a solvent that strongly interacts with the catalyst, such as alcohol, partially limits substrate conversion but drives the formation of the desired hydrogenolysis reaction products.

The reaction performed in 2-propanol yielded a high substrate conversion further suggesting a possible other role of the solvent in the reaction *via* a H-transfer reaction to the substrate.^44^ 2-propanol was therefore selected as the reaction solvent for the remainder of the work presented here.

Following the optimization of the reaction conditions and the solvent screening experiments, the materials with relatively lower Ni loadings, *i.e*., 5 and 10% were also applied in the hydrogenolysis of BPE. The results of this investigation are displayed in Table 4 and for comparison, results over the 20% Ni material are reshown.

**Table 4:** Investigation of the Catalysts in the Ni/ZSM-5 Series for the Hydrogenolysis of BPE under a H_2_ Atmosphere^a^

catalyst	BPE conv. (%)	toluene sel. (%)	phenol sel. (%)
5% Ni/ZSM-5	80.4	90.1	78.8
10% Ni/ZSM-5	99.1	98.7	55.7
20% Ni/ZSM-5	90.6	82.6	75.8

a Reaction conditions: BPE (50 mg), cat. (25 mg), solvent (50 mL), *t*: 2 h, *T*: 250 °C, IHP: 8.0 bar.

All investigated Ni-containing catalysts were efficient for the conversion of BPE to toluene and phenol under these conditions. The lowest substrate conversion was obtained over the 5% Ni-ZSM-5 catalyst (80.4%), while interestingly, the 10% Ni-ZSM-5 catalyst was more efficient than the 20% Ni-ZSM-5 catalyst for the promotion of the reaction. This increased reactivity might be attributed to the presence of highly reactive nickel nanoparticles with (111) exposed facets on the former and not on the latter (based on the observations made from the analysis of TEM images above).

Interestingly, computational publications have shown that activated hydrogen species are preferentially generated from the decomposition of alcohols over (111) exposed facets than over (100) and (110) facets.^45^ This was, in all cases, linked to the preferential cleavage of the alcohol O–H bond over that of the C–O bond over metallic (111) facets which was not observed over the other studied facets.^46^

This indicates that an improvement in the proposed hydrogen transfer reaction rate could explain the observed differences in reactivity between our catalysts with different exposed facets for BPE hydrogenolysis, with Ni (111) exposed facets favoring the hydrogenolysis of BPE through interaction with 2-propanol. The superior activity of (111) exposed facts for hydrogen addition reactions have also been demonstrated by means of DFT calculations by Wang et al.^47^ for hydrogenation of CO_2_ over Fe-based catalysts and by Ma et al.^48^ for the hydrogenation of gasoline over Pd-based catalysts.

Experimental results have also been published in the field supporting our reasoning for the observed relatively higher activity of the (111) exposed facets catalyst (*i.e*., the 10% Ni material) over the other materials. Campbell et al.^49^ proposed the lower activation energy of Pd(111) facets as the reason for this material’s higher reactivity in the hydrogenolysis of 1-butanol compared to that of a Pd(211) facets catalyst. Cui et al.^50^ have also concluded, using both experimental data and computational calculations, that the lower reaction energy barrier calculated for (111) exposed facets was the reason for the higher reactivity of catalysts in the hydrogenation of oxygen-containing compounds.

Interestingly, Goodman^51^ presented results that display an opposite trend with Ni-based catalysts having (100) exposed facets being more reactive for the hydrogenolysis of ethane than analogous Ni-based catalysts with (111) exposed facets, highlighting the importance of experimental data gathering on the effect of surface morphology on catalytic materials with applications in hydrogen-based reaction.

**Figure 7:**
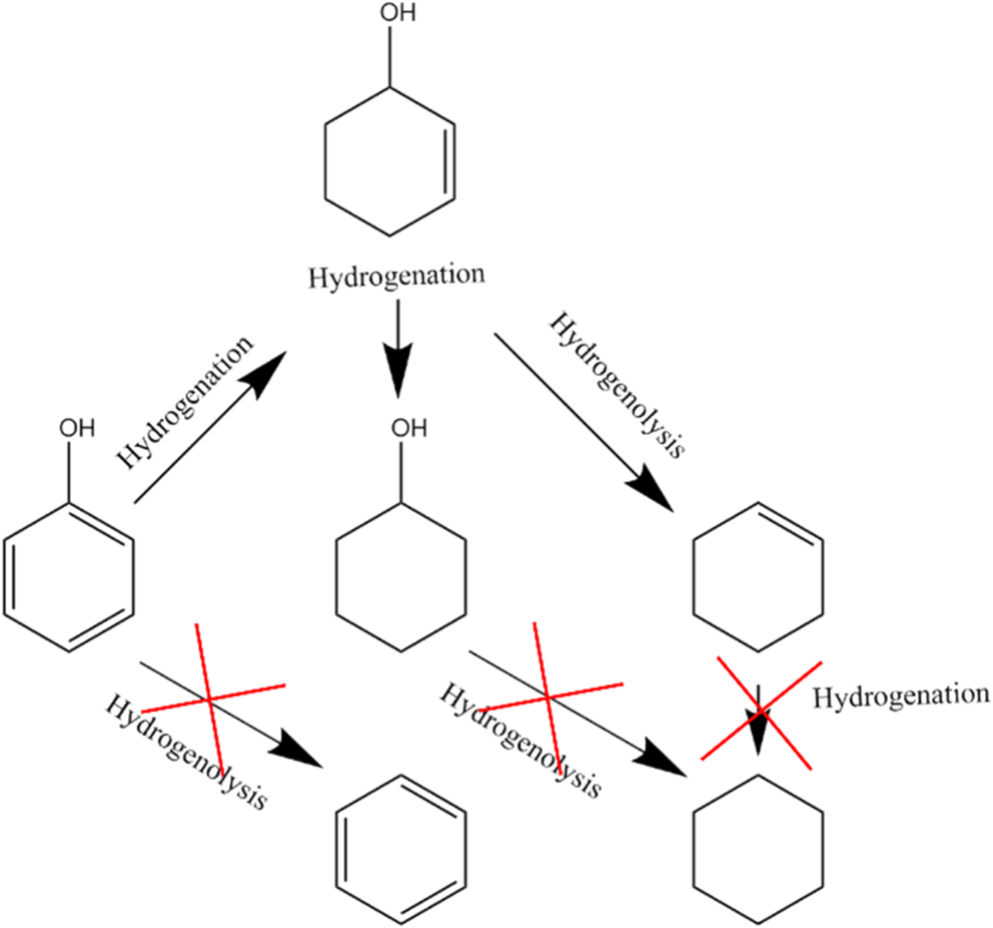
Proposed reaction scheme for the further conversion of phenol to cyclic compounds over bifunctional acid-metal catalysts.

The relatively low selectivity to phenol obtained in the reaction performed over the 10% Ni-ZSM-5 catalyst (55.7%) was attributed to the further *in situ* conversion of this product to a range of cyclic compounds (a reaction scheme is proposed in Figure 7).

This reaction scheme is based on a probe qualitative reaction performed using phenol as a substrate under similar reaction conditions. During this reaction, phenol was partially converted to cyclohexene, cyclohexenols (*i.e*, cyclohex-1-en-1-ol and cyclohex-2-en-1-ol and cyclohex-3-en-1-ol) and cyclohexanol. Neither benzene nor cyclohexane were detected as reaction products, indicating that the hydrogenation of the benzene ring occurred as the first reaction step.

These results were attributed to interactions between the electron pairs of the oxygen in phenol with the empty *d* band orbitals of the Ni nanoparticles, allowing for the efficient coordination of the compound on the catalyst, eventually favoring the hydrogenation of the benzene ring.

The hydrogenated product can also be further converted to either cyclohexene *via* hydrogenolysis or to cyclohexanol *via* further hydrogenation. No further conversion of these products to cyclohexane was noted.

These reactions are expected to have occurred to a certain extent over all catalysts and under all investigated reaction conditions, in all cases generating low levels of cyclic compounds and decreasing the measured selectivities to phenol. In the case of the reaction performed over the 10% Ni catalyst, the low selectivity to phenol might be attributed to a longer residence time of this compound in solution, which is an indication that the quantitative conversion of BPE may have occurred before the 2-h mark. This further suggests the high efficiency of this catalyst for the conversion of the model compound. This proposed reaction scheme has rarely been reported and could be of interest for the design of reactions aiming for the production of cyclic compounds, typically for the generation of biobased jet fuels.

Conversely, high selectivities to toluene (>80.0%) were obtained for all reactions presented in this work, indicating the relative stability of this product under these reaction conditions. The proposed rationale for the distinct fate of the two main reaction products could be of significance for hydrodeoxygenation reactions (*i.e*., reactions generating oxygen-free products) as well as for the design of catalysts within biorefining lines aimed at the generation of phenolic compounds. In the latter case, overly reactive catalysts generating oxygen-free compounds would not be beneficial and the results presented here suggests that a trade-off between conversion of the substrate and stability of the reaction products must be included in the design of catalytic materials for industrial-scale applications.

Following the hypothesis that 2-propanol may play a role as a H-transfer solvent to promote the hydrogenolysis reaction,^52^ the experiments discussed above were repeated under an inert atmosphere (in Ar) under otherwise comparable reaction conditions. The results of this investigation are displayed in Table 5.

**Table 5:** Investigation of the Catalysts in the Ni/ZSM-5 Series for the Hydrogenolysis of BPE under an Ar Atmosphere^a^

catalyst	BPE conv. (%)	toluene sel. (%)	phenol sel. (%)
5% Ni/ZSM-5	49.9	82.5	67.9
10% Ni/ZSM-5	99.1	92.8	63.6
20% Ni/ZSM-5	98.7	100	70.8

a Reaction conditions: BPE (50 mg), catalyst (25 mg), 2-propanol (50 mL), *t*: 2 h, *T*: 250 °C, IArP: 8.0 bar.

Over both the 10 and 20% Ni-ZSM-5 catalysts almost complete substrate conversions were obtained in 2-propanol, in absence of gaseous H_2_. This confirms that the solvents could be activated (generating acetone as a byproduct) by the Ni-containing catalysts and 2-propanol acted as a H-transfer reactant to promote the hydrogenolysis reaction. The formation of acetone during these reactions was confirmed by GC analysis but the level of formation of this compound could not be quantified due to important overlapping in peaks of acetone and 2-propanol.

Interestingly, the selectivities to both toluene and phenol tend to be higher for reactions performed under an Ar atmosphere over all materials. This indicates that the activation of external hydrogen over the Ni-ZSM-5 catalysts favors undesired parallel reactions as well as the hydrogenolysis route and that this can be limited by performing the reaction under an inert atmosphere. The use of an inert atmosphere is also intrinsically safer (and potentially cheaper), which can be of significant interest in the context of biorefining.

However, a significant drop in substrate conversion (from 80.4 to 49.9%) over the catalyst with the lowest Ni loading (5%) was noted with respect to the corresponding reaction performed under a H_2_ atmosphere. Over this relatively low loaded Ni catalyst, it is clear that both the H_2_ activation and the 2-propanol H-transfer processes played parts in the promotion of the hydrogenolysis reaction described in Table 4.

### 3.5 Postreaction Characterization

Following reactions under both atmospheres (*i.e*., H_2_ and Ar) all catalysts were collected by filtration and characterized by p-XRD, TEM and TGA to investigate possible structural modifications or carbonaceous deposit formation during reaction.

The p-XRD postreaction characterization of the catalysts used in the reaction under a H_2_ atmosphere (see Figure S5) confirmed the stability of the crystalline structure of zeolite and showed that the Ni nanoparticles had sintered on the material.

**Figure 8:**
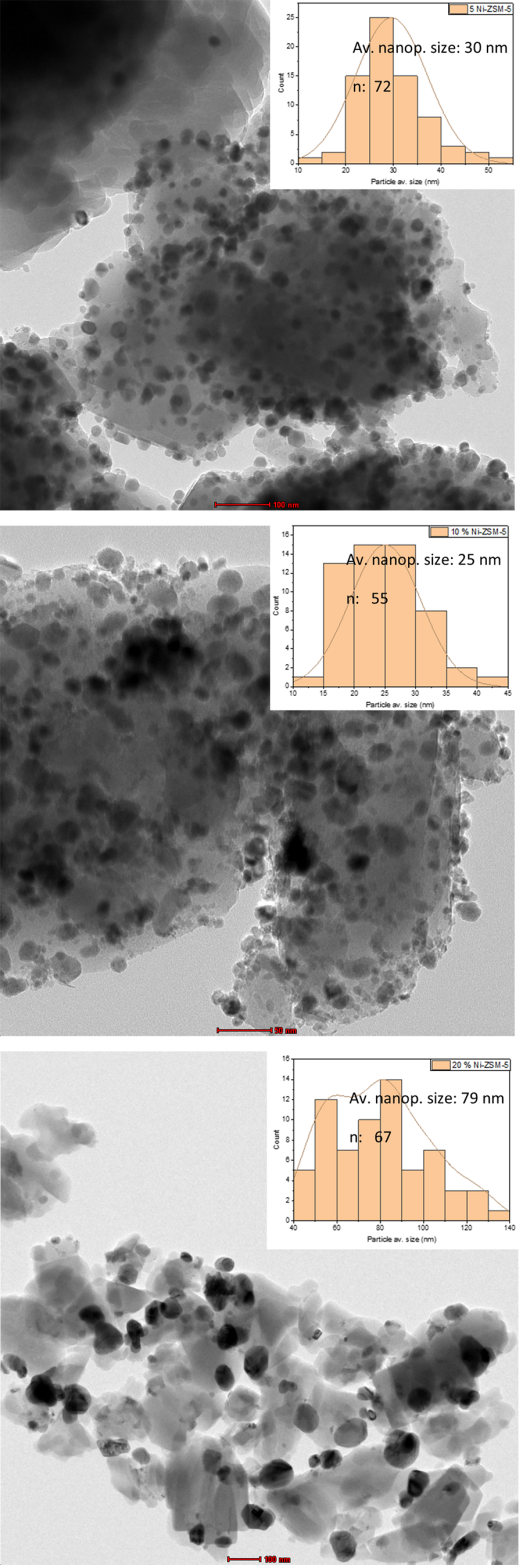
TEM images of the postreaction Ni-ZSM-5 series catalysts following reactions performed under a H_2_ atmosphere.

This was confirmed by a lower F.W.H.M. of the Ni peaks in the profiles of the used materials with respect to the corresponding peaks in the prereaction profiles. This was also confirmed by postreaction TEM analysis (see Figure 8). This confirms particle sintering under reaction conditions and this effect has previously been reported for similar materials under similar conditions.^53^

The average Ni particle size increased from 18 to 30 nm on the 5% Ni catalyst, from 21 to 25 nm on the 10% Ni catalyst and from 29 to 79 nm on the 20% Ni catalyst. Interestingly, on the images of the 10% Ni material a decrease in prevalence of the tetrahedral shaped nanoparticles was observed, indicating that the morphology of these may not be stable under the studied reaction conditions. This result coupled with the reactivities discussed above highlight the importance of postreaction morphological analysis of supported nanoparticles in deactivation studies.

The p-XRD postreaction characterization of the 5% Ni and 20% Ni materials used for reaction under an Ar atmosphere followed the same trend (see Figure S6) with however a significantly lower levels of particle sintering noted. This was also confirmed by postreaction TEM analysis (see Figure S7). In this case, a lower prevalence of tetrahedral shaped Ni nanoparticles was also noted postreaction on the images of the 10% Ni material. It has been reported that under certain reaction conditions the morphology of nanoparticles with well-defined shapes can be altered due to oxidation reactions.^54^ This is an indication that the tetrahedral Ni nanoparticles are not stable under either of the atmospheres under the studied reaction conditions. Interestingly, another phenomenon was observed in the profile of the 10% Ni material following the reaction under Argon.

**Figure 9:**
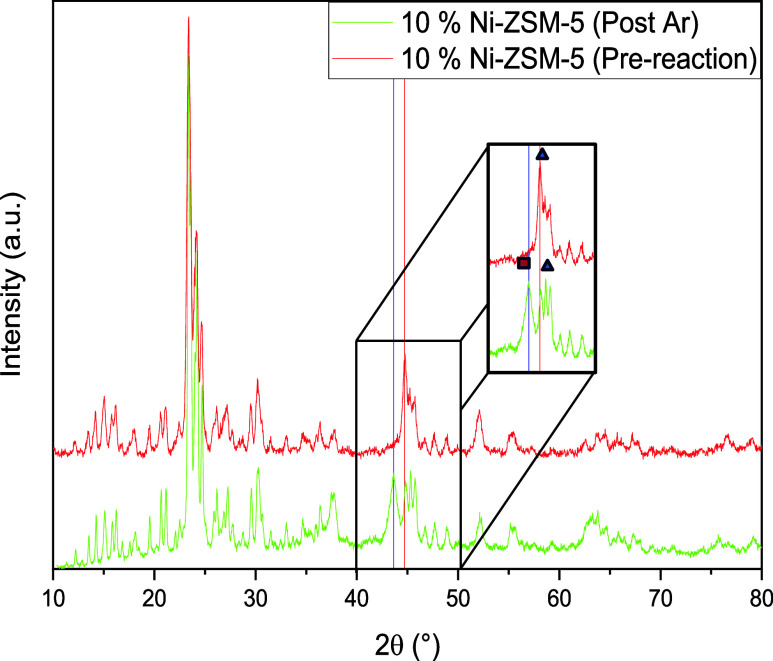
p-XRD profiles of the 10% Ni-ZSM-5 catalyst before and after reaction under an Ar atmosphere. Ni:blue triangle up solid; NiO:red box solid.

In this case, p-XRD evidence for the presence of NiO particles was clearly detected (see Figure 9). The formation of this oxide can only arise from a substrate-catalyst interaction during the partial further conversion of phenol to cyclic compounds during the reaction as this molecule (along with the substrate) was the only source of oxygen in the reaction mixture. This is also in-line with the relatively lower selectivity to phenol obtained for this reaction. This suggests that the tetrahedral shaped nanoparticles observed only on this material are more prone to oxidation under these reaction conditions than the spherical particles (*i.e*., the nanoparticles present on both other catalysts) on which no oxidation to NiO species was detected.

The *in situ* oxidation of the Ni nanoparticles was not noted in the postreaction p-XRD profile of the same material used for the same reaction performed under H_2_. There is however no reason to believe that this oxidation step did not occur also under these conditions, indicating that the H_2_ here converted any formed NiO species back to metallic Ni nanoparticles. This suggestion is supported by similar results from Alonso et al.^55^ and Yung et al.^56^

From the correlation of both the TEM and p-XRD postreaction analysis of the materials, it is proposed that the mechanism responsible for the lower levels of tetrahedral Ni nanoparticles observed postreaction relates to their *in situ* oxidation that can only be noticed for reaction performed under an inert atmosphere. However, no other work mentioning this phenomenon could be found in literature, we suggest that *in situ* characterization methods would bring further indications on the oxidation/reduction events occurring and would be of fundamental interest in the field of hydrogen-based catalytic reactions.

**Figure 10:**
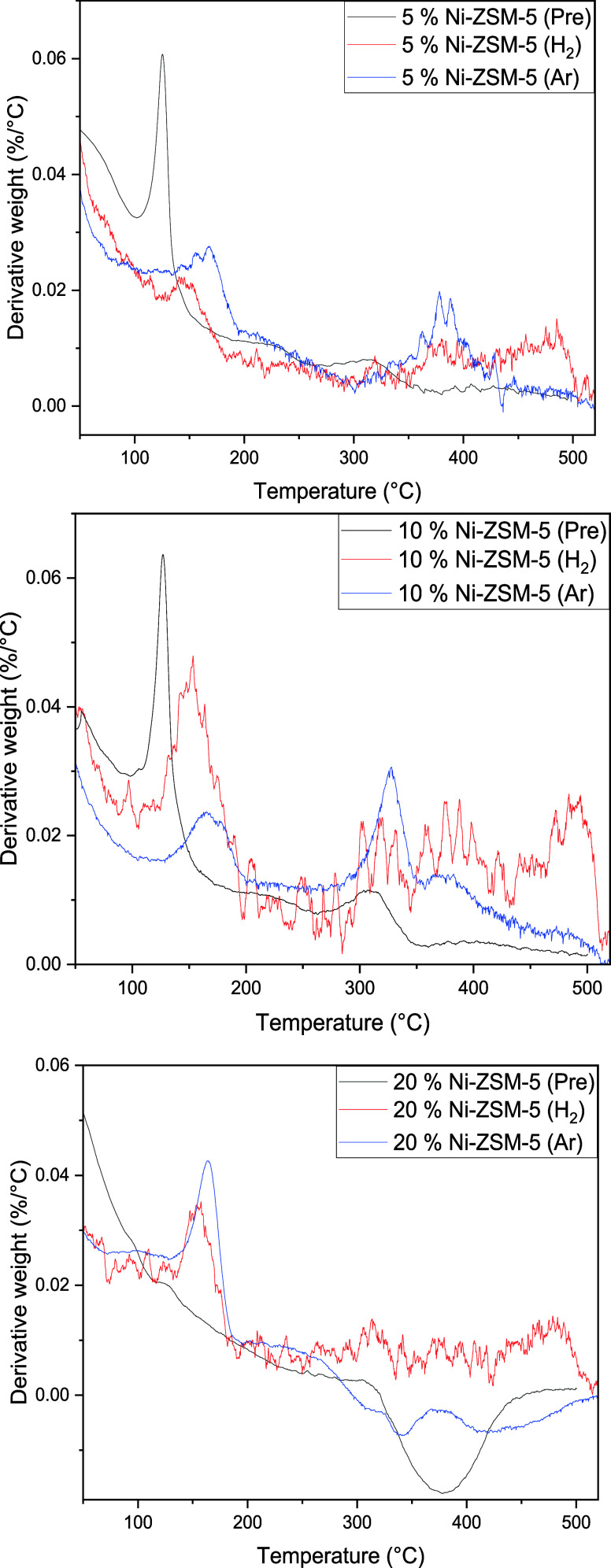
DTGA profiles for all three materials pre- and postreaction, 5% Ni (top), 10% Ni (middle) and 20% Ni (bottom).

TGA measurements were performed both before and after reaction on all materials to investigate the possible formation of carbonaceous deposits on the catalysts during reaction. The DTGA profiles of all materials used for reaction under both H_2_ and Ar atmospheres are shown in Figure 10.

A significant weight loss event was noticed around 100 °C for both the 5% Ni and 10% Ni materials prereaction, this was attributed to the desorption of water from the porous H-ZSM-5 support. As expected, this was not seen in any of the postreaction TGA profiles (the postreaction samples were collected and dried prior to measurement). However, other weight loss events at higher temperatures were noticed in the TGA patterns of all postreaction materials. The temperatures of these events (300–500 °C) indicate the removal of carbonaceous deposits from the catalysts.

A first weight loss occurs around 150 °C, this was noticed for all materials and under both atmospheres, however in the TGA profiles of the materials used in reactions performed under an Ar atmosphere this peak is noticed at slightly higher temperatures in all cases. This could be an indication that the nature of the carbonaceous deposits formed during the reaction was influenced by the atmosphere used. The peaks for materials used under the different atmospheres are comparable in intensities from both the 5% Ni and 20% Ni materials, but a significantly higher formation of deposit was noted on the 10% Ni catalyst used under a H_2_ atmosphere than on the one used an Ar atmosphere.

Two separate, high temperature, weight loss events were observed in the pattern corresponding to the postreaction 5% Ni (H_2_) material, around 350 °C and above 450 °C. The latter event was not seen in the pattern of the 5% Ni (Ar) indicating the formation of less, and easier to calcine, carbonaceous deposits under these reaction conditions. A similar observation was made for the postreaction 10% Ni materials with however a slight shift of the first peak to a lower temperature (around 325 °C).

A Ni nanoparticle oxidation event was noticed at high temperature (starting around 300 °C) in the prereaction 20% Ni material and not over the other two materials. This could not be explained but hinders the in-depth analysis of weight loss events in the patterns of the corresponding postreaction materials. It was however noted that larger carbonaceous deposits had formed on the catalyst used under a H_2_ atmosphere than on the one used under an Ar atmosphere, similar to what was observed for the other two materials.

Overall, lower carbonaceous deposition was noted for materials used under an Ar atmosphere than for the analogue materials used under a H_2_ atmosphere, indicating that the latter tends to promote the formation of these deposits. The deposits formed on the catalysts used under an Ar atmosphere could also be calcined at relatively lower temperatures (suggesting they were different). Again, this phenomenon is understudied and more systematic focus on the nature of the carbonaceous deposit formed during reaction under different atmosphere would be of interest to the field. However, it is clear that this (along with the morphological changes to the Ni particles highlighted above) would prohibit the facile recycling of these materials in the absence of regeneration steps. Furthermore, no measurements of whether Ni was leached from the materials during reaction, and clearly, were these catalysts to be employed industrially, such measurement would be needed.

These postreaction characterization results indicate that these catalysts are not sufficiently stable for industrial-scale practical applications under the studied reaction conditions, however the valorization of native lignin will require reactions conditions that may have important differences with the ones studied here. Further work will be required for the preparation of comparable Ni-ZSM-5 catalysts with improved stability under hydrogenolysis promoting conditions, using more advanced model compound substrates and, eventually, lignin itself.

We believe that the results presented in this work form a solid ground in that optic, with the identification of solvents that allow for the use of inert atmosphere as well as relatively mild reaction conditions and the identification of a metal loading-related effect on the supported nanoparticles morphology, that allows for the use of relatively less metal while improving the reaction rate.

## 4 Conclusions

This study has looked at the characterization and catalytic properties of a family of Ni-ZSM-5 catalysts prepared with different nominal metal loadings (5, 10 and 20%) in the hydrogenolysis of a lignin model compound (benzyl phenyl ether) under different reaction conditions. Higher reaction temperatures and/or higher H_2_ pressures led to higher substrate conversions without having any significant effect of the selectivity to the products of interest. A solvent screening showed a key role of 2-propanol in the hydrogenolysis reaction through both hindrance due to solvent-metal interactions favoring the hydrogenolysis reaction pathway and through a H-transfer reaction promoting the hydrogenolysis reaction even under an inert (Ar) atmosphere. This confirmed that two separate hydrogen sources cooperated in the promotion of the hydrogenolysis of benzyl phenyl ether, *i.e*., the dissociation of H_2_ and the H-transfer reaction from 2-propanol.

Of the three Ni-ZSM-5 catalysts, a 10% Ni loaded material was found to be the most efficient for promoting the reaction under H_2_. This was attributed to the presence of tetrahedral nanoparticles with Ni (111) exposed facets on this material which are known to activate H_2_. It was however noted that at comparable conversions (*i.e*., above 90%) the selectivities to phenol and toluene were higher over the 20% Ni catalyst than over the 10 Ni % catalyst. Furthermore, in the absence of H_2_, the 10% Ni material was prone to *in situ* oxidation to NiO while the other two were not, a further effect of the tetrahedral nanoparticles.

Postreaction characterization of the materials also provided insight on the impact on the two different reaction atmospheres used in this study. Both conditions resulted in Ni particle sintering and carbonaceous deposition on all materials, but both effects were more pronounced under H_2_ and clearly this may hinder the reusability of these catalysts.

Ni-ZSM-5 materials prepared *via* a simple excess impregnation method were shown to be an interesting alternative to commonly used noble-metal containing analogues for this reaction of potential interest in lignin biorefining.

## Supporting Information Available

Image of the reactor used and details of its operation; XRD profiles of the materials taken during the preparation process; a reaction scheme for the conversion of 1 butanol to 1,1 dibutoxybutane; a GC profile of the reaction mixture following a reaction over the 20% Ni catalyst; XRD patterns of Ni-ZSM-5 catalysts and after reactions performed under H_2_; XRD patterns of all Ni-ZSM-5 catalysts before and after reactions under Ar; TEM images of the postreaction Ni-ZSM-5 catalysts following reactions under Ar (PDF)
