# Supplementary material for: Study of Ni-ZSM-5 Catalysts in the Hydrogenolysis of Benzyl Phenyl Ether: Effects of Ni Loading, Morphology, and Reaction Conditions
Source: ACS Omega. 2025 Mar 17;10(12):12306–18. doi: 10.1021/acsomega.4c11273 (PMC11966267; doi:10.1021/acsomega.4c11273)
Supplement: Supplementary file 1 — ao4c11273_si_001.pdf [file ao4c11273_si_001.pdf]

A study of Ni-ZSM-5 catalysts in the hydrogenolysis of benzyl phenyl ether: Effects of Ni loading, morphology and reaction conditions.

Raphaël Abolivier [a], *Hans-Georg Eckhardt* [a], *James A. Sullivan* [a]*

Email address of the corresponding author: james.sullivan@ucd.ie

[a] University College Dublin, School of Chemistry, Belfield, Dublin 4, D04 V1W8, Dublin, Ireland.

Supporting information

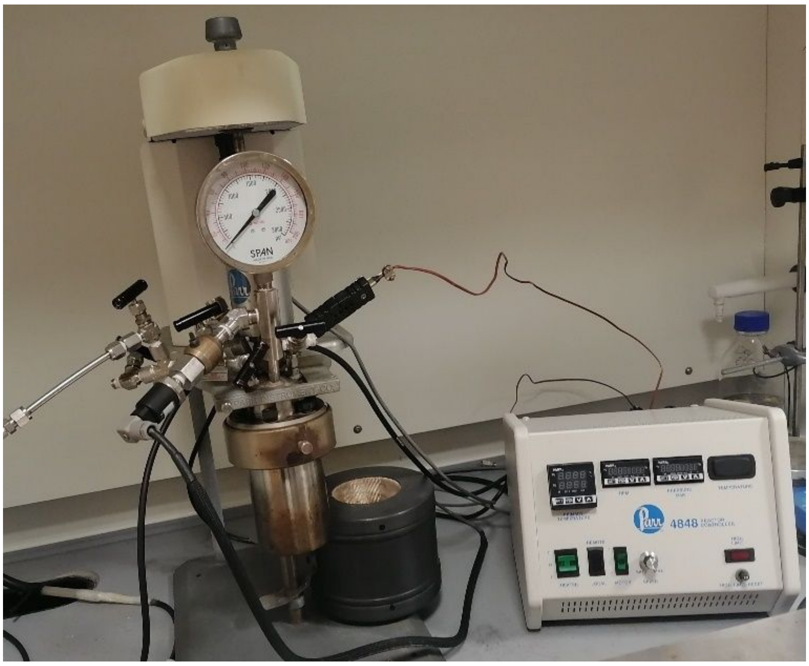

Fig. S1. Photograph of the used Parr reactor setup.

All reactions discussed in this work were performed in a high pressure and stirred Parr 300 mL reactor (see Fig. S1). This 300 mL bench top 4566 mini reactor can be used at temperatures up to 350 °C and pressures up to 200 bar. It is equipped with a mechanical stirrer (up to 800 rpm), a fixed thermocouple for temperatures measurements, a pressure gauge, and a heating mantle. This reactor is connected to a 4848 Parr reactor controller (on the right in Fig. S1) that enables control over the reaction temperature (through a connection to the reactor's thermocouple), independent pressure measurement (including a high-pressure limit control, for safety) and control over the stirrer rotation speed. The reactor was connected to the relevant gas lines through the built-in gas inlets. The hydrogen gas line was equipped with a flashback arrestor to prevent any danger of propagation in the case of an explosion in the vessel. In a typical reaction, a solution composed of the appropriate amount of reactant and catalyst in the chosen solvent was prepared in a beaker and transferred to the stainless-steel vessel. This vessel was then mounted on the reactor and air-tightened using two metallic split-rings with captive compression ring and bolts. The reactor was flushed three times with hydrogen to remove oxygen from the vessel during the reaction (to prevent any reaction between hydrogen and oxygen at elevated temperatures). Following this step, the vessel was pressurised with hydrogen gas to the desired initial hydrogen pressure (the control over the inlet pressure was performed directly using the gas cylinder regulator valve). The mantle was raised to the correct position and parametrized using the reactor controller. The stirring was started and the t_0 of the reaction was considered to be once the solution reached the expected reaction temperature (ranging between 200 °C and 300 °C). After the desired reaction time had elapsed, the heating and stirring were switched off, the mantle lowered, and the vessel rapidly cooled down to room temperature using iced water. At this stage,

the reactor was depressurised by opening the gas outlet valve and the vessel disconnected from the reactor. An aliquot was collected directly from the vessel using a syringe (equipped with a filter) and transferred to a GC vial for characterization.

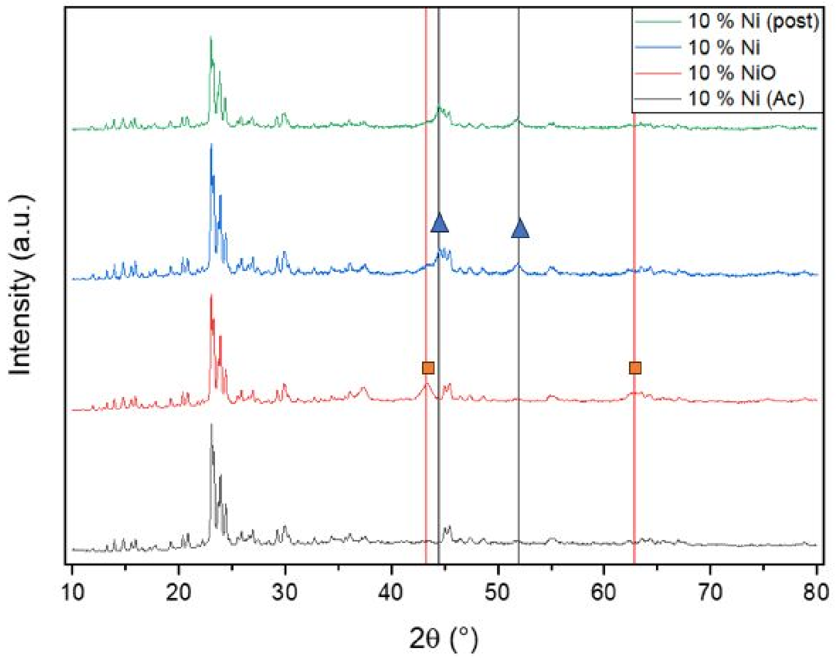

Fig. S2. p-XRD profiles of the 10 % Ni-ZSM-5 material at different stages of the preparation protocol and post-reaction (2θ: 5 ° to 80 °, △ Ni ; □ NiO). The pattern labelled "10 % Ni(Ac)", corresponds to the material obtained after the impregnation and drying steps, the "10 % NiO" pattern is that of the material post-calcination in air, the "10 % Ni" pattern is that of the reduced material under 3 % H_2/Ar atmosphere and the "10 % Ni (post)" pattern is that of the post-reaction material.

In order to investigate the effect of each preparation step on the prepared material's structure, p-XRD patterns were recorded of the 10 % Ni/ZSM-5 material after each preparation step to evaluate the evolution of the Ni crystalline structure. Another pattern of this material was recorded post-reaction to probe for any deactivation effects. The corresponding profiles are shown in Fig. S.2. No peaks corresponding to either metallic (△ and black vertical lines in Fig. S2) or oxidic (□ and red vertical lines in Fig. S2) Ni could be detected on the pattern of the "10 % Ni (Ac)" material. All peaks in this profile were solely attributed to reflections arising from the H-ZSM-5 support's crystalline structure. The deposited nickel acetate was oxidized, as can be concluded from the presence of reflections attributed to NiO in the profile of the "10 % NiO" material (2θ: 43.3° and 62.8° corresponding to NiO (200) and (220) reflections, respectively)[1]. No peaks corresponding to the metal-oxide phase remain in the profile of the "10 % Ni" material, indicating that the supported nanoparticles were successfully reduced. The "10 % Ni (post)" pattern is of the material collected after having been used for the investigated reaction under a H_2 atmosphere. No significant differences in peak position between this profile and the "10 % Ni" pattern were noticed. However, the Ni-related peaks have slightly higher intensity (and lower FWHM) in the post-reaction profile than in the pre-reaction profile indicating particle sintering during the reaction. The post-reaction pattern of the 10 % Ni-ZSM-5 used for the reaction performed under an Ar atmosphere is discussed in the main manuscript as it presents specificities of high interest.

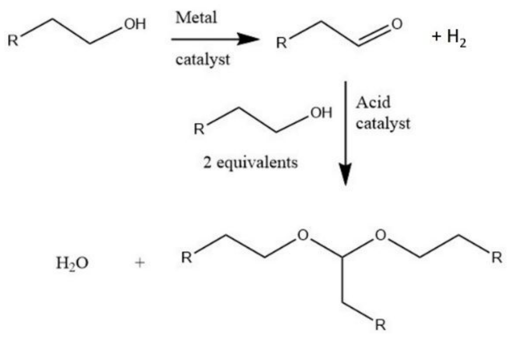

Fig. S3. Proposed reaction scheme for the metal/acid catalysed conversion of 1-butanol to 1,1-dibutoxybutane (R: CH_3CH_2-).

A proposed reaction scheme for the conversion of 1-butanol to 1,1-dibutoxybutane is shown in Fig. S3[2]. The first step is the oxidative dehydrogenation of the solvent (*i.e.*, forming 1-butanal) occurring on metal sites[3]. 1,1-dibutoxybutane was then formed by the condensation of two molecules of the alcohol and a molecule of the aldehyde, occurring over an acid site[4].

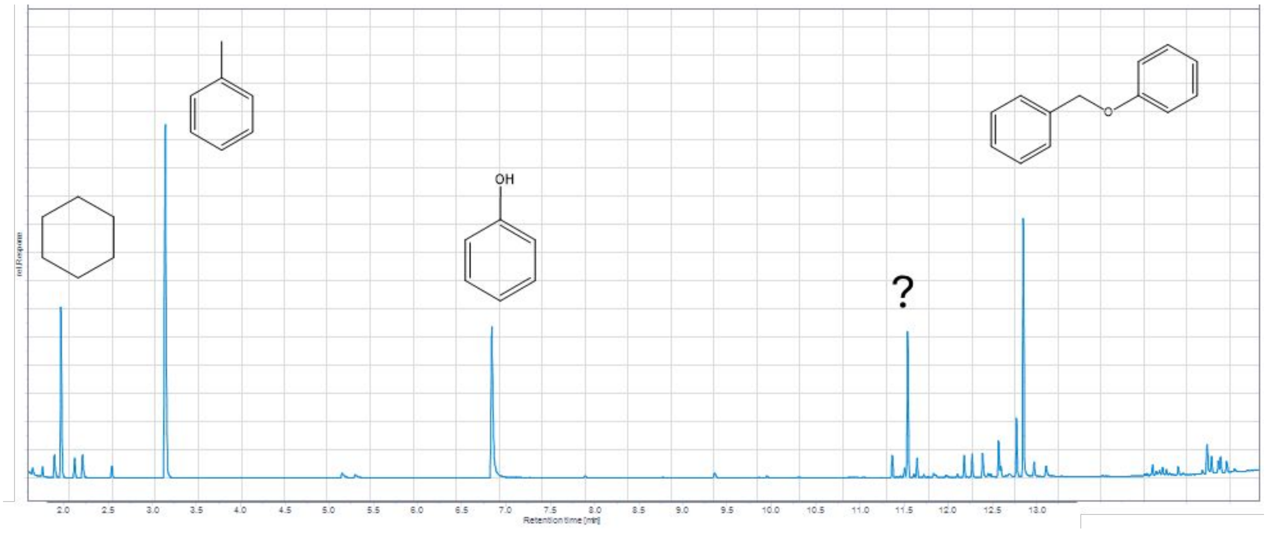

Fig. S4. Gas chromatography profile of the reaction mixture following a reaction over the 20 % Ni-ZSM-5 catalyst in pentane.

The GC profile showing the analysis of the reaction performed in pentane is shown in Fig. S4. It was found that whilst both toluene and phenol were generated in large quantities both cyclohexane and another, unidentified compound (with a retention time: 11.7 min, marked as "?" in Fig. S5), were also reaction products. Based on the relatively high retention time for the latter product, it is proposed that it could be a partially hydrogenated version of the substrate. These compounds were not found in any mixture from reactions performed in the other selected solvents. The production of these compounds is the reason for the relatively low selectivities obtained for both toluene and phenol for this reaction.

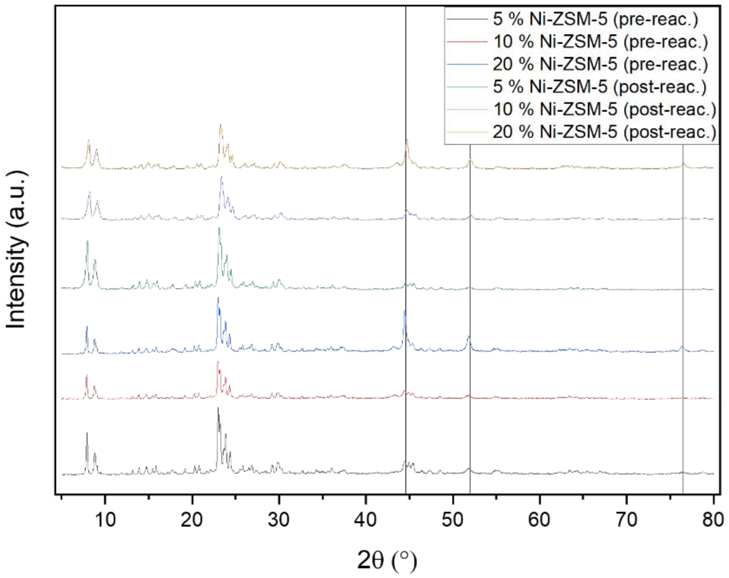

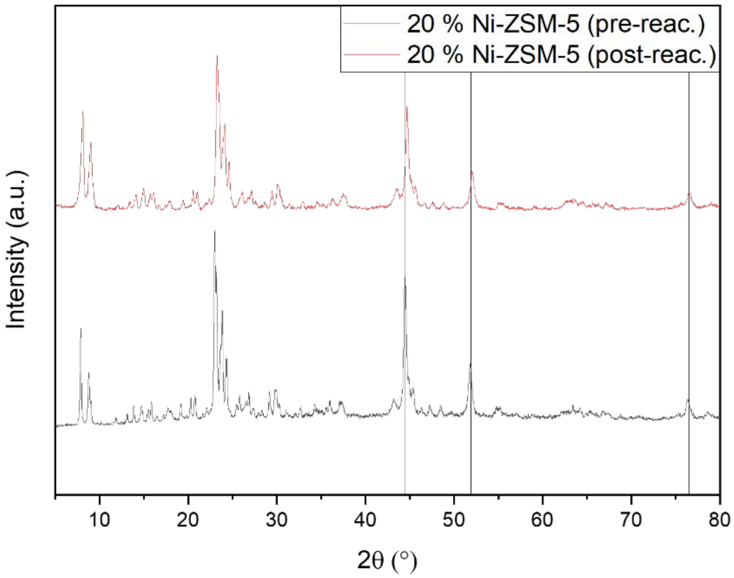

Fig. S5. p-XRD patterns of all Ni-ZSM-5 catalysts (top) before (5 %: 10 % and 20 % in black, red and blue, respectively) and after (5 %: 10 % and 20 % in green, purple and orange, respectively) reactions performed under H_2 atmosphere. The p-XRD patterns of the 20 % Ni-ZSM-5 catalyst (black: before; red: after) are shown as stand-alone (bottom) for clarity.

Following the reactions performed under a H_2 atmosphere the three catalysts were collected by filtration and characterized again using p-XRD, see in Fig S5, no changes in diffraction patterns (regarding peak positions) were observed following the reaction in any of the profile corresponding to these materials. The p-XRD patterns are shown for all samples (top) and for the 20 % Ni-ZSM-5 only (bottom) for ease of comparison. This indicates the stability of the catalysts under these conditions, no phase transition or oxidation of the Ni nanoparticles occurred. However, the peaks corresponding to diffractions arising from the Ni structures had higher intensities (and lower FWHM) in the profiles of the post-reaction materials. This was attributed to the sintering of the supported Ni nanoparticles during the reactions.

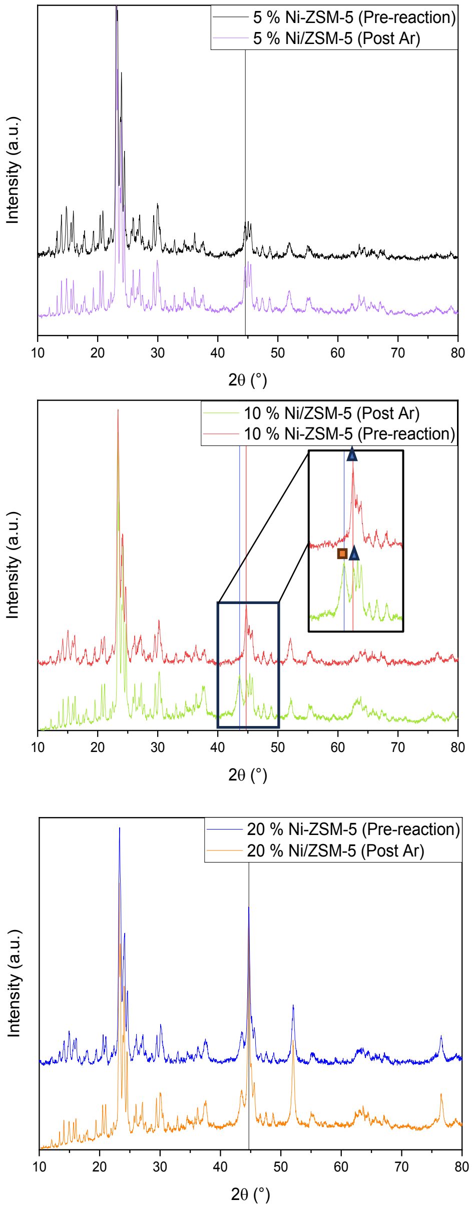

Fig. S6. p-XRD patterns of all Ni-ZSM-5 catalysts before and after reactions performed under an Ar atmosphere (5 % Ni/ZSM-5 (top); 10 % Ni/ZSM-5 (middle, △: Ni0 and □: NiO) and 20 % Ni/ZSM-5 (bottom)).

The catalysts used for the reactions performed under an Ar atmosphere were collected by filtration and characterized by p-XRD. The p-XRD patterns corresponding to these post-reaction materials are shown in Fig. S6. It can be seen that the profiles of both the 5 % Ni-ZSM-5 and 20 % Ni-ZSM-5 were very similar to one another before and after reaction, with the exception of the intensity of the Ni-related peaks (and therefore the F.W.H.M. of these peaks), as was seen above in the post-reaction p-XRD patterns of the materials used to promote the reaction under H_2 atmosphere. This indicates that no oxidation of the metals occurred during the reactions but that some nanoparticle sintering had taken place. In the profile of the 10 % Ni-ZSM-5 post-reaction material a peak not observed in the profile of the corresponding pre-reaction material can be seen (2θ: 43.6 °, red horizontal line in Fig. S6). This peak is attributed to a (111) reflection from NiO. The peak corresponding to the (111) reflection of the Ni metal is also relatively lower in intensity in the pattern of the post-reaction material (2θ: 44.7 °). This suggests that a proportion of the metal nanoparticles deposited on this material are oxidized in-situ during the reaction performed under Ar. This is an important potential deactivation mechanism as the H-transfer reaction is catalysed by metallic species and not by metal-oxide species.

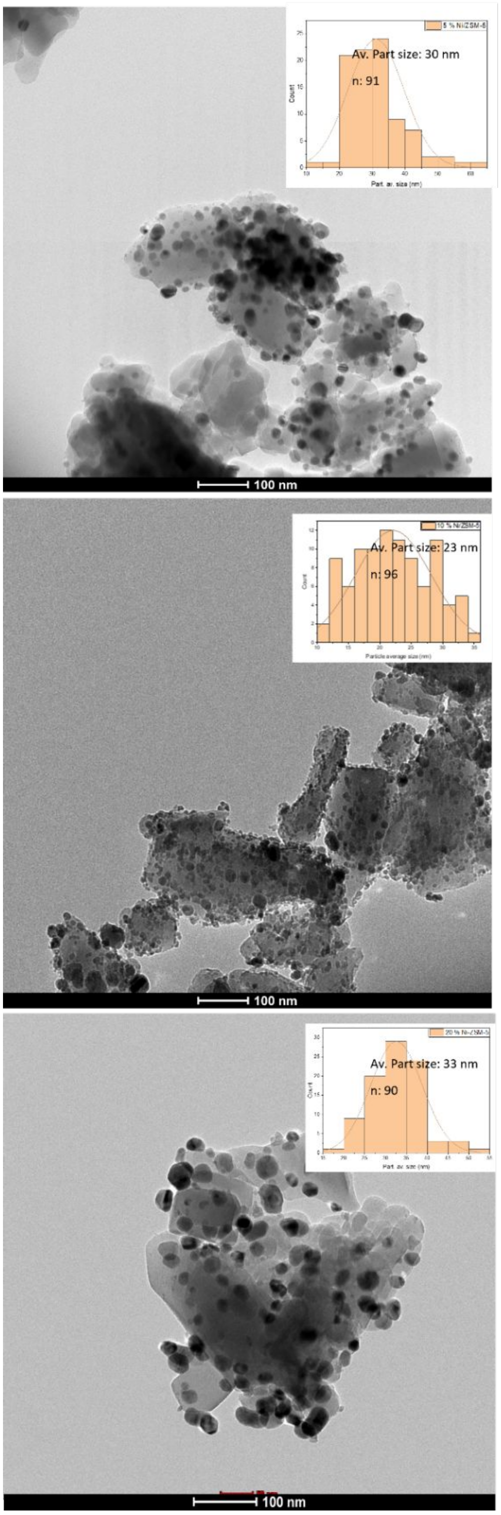

Fig. S7. TEM images of the post-reaction Ni-ZSM-5 series catalysts following reactions performed under an Ar atmosphere.

The TEM images of the materials following the reaction under Ar are shown in Fig. S7. It can be seen that nanoparticle sintering also occurred for the catalysts used in the reactions performed under an Ar atmosphere. Interestingly, the average nanoparticle sizes observed on the TEM images of the two post-reaction 5 % Ni-ZSM-5 catalysts (*i.e.*, under either H_2 or Ar atmospheres) are similar to one another (*ca.* 30 nm). This is also the case for both 10 % Ni/ZSM-5 materials (*ca.* 24 nm). This is however not the case for the 20 % Ni/ZSM-5 material for which the observed sintering is more significant on the material used for the reaction under H_2 (79 nm) than for the one under Ar (33 nm).

(1) Richardson, J. T.; Scates, R.; Twigg, M. V. X-ray diffraction study of nickel oxide reduction by hydrogen. *Applied Catalysis A: General* **2003**, *246* (1), 137-150.
(2) Uemura, S.; Yoshida, T.; Koga, M.; Matsumoto, H.; Yang, X.; Shinohara, K.; Sasabe, T.; Hirai, S. Ink degradation and its effects on the crack formation of fuel cell catalyst layers. *Journal of the Electrochemical Society* **2019**, *166* (2), F89.
(3) Falah, I. I.; Pradipta, M. F.; Rinaldi, A. L.; Trisunaryanti, W. Synthesis 1, 1-Dibutoxybutane from Single Reagent of n-Butanol Using Cr/Activated Carbon Catalyst. *Indonesian Journal of Chemistry* **2020**, *20* (5), 1018-1024.
(4) Yoeswono, Y.; Triyono, T.; Falah, I. Catalytic activity of Mn/AC catalyst on direct synthesis of 1, 1-dibutoxybutane from 1-butanol. In *AIP Conference Proceedings*, 2016; AIP Publishing: Vol. 1755.
